# Dolerite Fines Used as a Calcium Source for Microbially Induced Calcite Precipitation Reduce the Environmental Carbon Cost in Sandy Soil

**DOI:** 10.3389/fmicb.2020.557119

**Published:** 2020-09-08

**Authors:** Carla C. Casas, Alexander Graf, Nicolas Brüggemann, Carl J. Schaschke, M. Ehsan Jorat

**Affiliations:** ^1^School of Applied Sciences, Abertay University, Dundee, United Kingdom; ^2^Institute for Bio- and Geosciences, IBG-3: Agrosphere, Forschungszentrum Jülich, Jülich, Germany; ^3^School of Computing, Engineering and Physical Sciences, University of the West of Scotland, Paisley, United Kingdom

**Keywords:** CO_2_ sequestration, CO_2_ emissions, MICP, calcium-rich silicate rock, basaltic quarry fines, weathering, calcite, pedogenic carbonates

## Abstract

Microbial-Induced Calcite Precipitation (MICP) stimulates soil microbiota to induce a cementation of the soil matrix. Urea, calcium and simple carbon nutrients are supplied to produce carbonates via urea hydrolysis and induce the precipitation of the mineral calcite. Calcium chloride (CaCl_2_) is typically used as a source for calcium, but basic silicate rocks and other materials have been investigated as alternatives. Weathering of calcium-rich silicate rocks (e.g., basalt and dolerite) releases calcium, magnesium and iron; this process is associated with sequestration of atmospheric CO_2_ and formation of pedogenic carbonates. We investigated atmospheric carbon fluxes of a MICP treated sandy soil using CaCl_2_ and dolerite fines applied on the soil surface as sources for calcium. Soil-atmosphere carbon fluxes were monitored over 2 months and determined with an infrared gas analyser connected to a soil chamber. Soil inorganic carbon content and isotopic composition were determined with isotope-ratio mass spectrometry. In addition, soil-atmosphere CO_2_ fluxes during chemical weathering of dolerite fines were investigated in incubation experiments with gas chromatography. Larger CO_2_ emissions resulted from the application of dolerite fines (116 g CO_2_-C m^–2^) compared to CaCl_2_ (79 g CO_2_-C m^–2^) but larger inorganic carbon precipitation also occurred (172.8 and 76.9 g C m^–2^, respectively). Normalising to the emitted carbon to precipitated carbon, the environmental carbon cost was reduced with dolerite fines (0.67) compared to the traditional MICP treatment (1.01). The carbon isotopic signature indicated pedogenic carbonates (δ^13^C_av_ = −8.2 ± 5.0‰) formed when dolerite was applied and carbon originating from urea (δ^13^C_av_ = −46.4 ± 1.0‰) precipitated when CaCl_2_ was used. Dolerite fines had a large but short-lived (<2 d) carbon sequestration potential, and results indicated peak CO_2_ emissions during MICP could be balanced optimising the application of dolerite fines.

## Introduction

Microbial-Induced Calcite Precipitation (MICP) is an emerging soil stabilisation technique based on the biomediated precipitation of the mineral calcite within soil by urea hydrolysis. Urease producing microorganisms are stimulated to catalyse urea hydrolysis into carbon dioxide (CO_2_) and ammonia (NH_3_). This reaction raises the soil pH leading to a shift in dissolved inorganic carbon (DIC) speciation towards carbonates which precipitate in the presence of divalent cations (Ca, Mg, Fe) as carbonate minerals. Carbonate minerals form on and accumulate around soil grain surfaces and microorganisms, which act as nucleation sites (Gat et al., 2014), and induces a cementation of the soil structure. When applied appropriately, MICP reduces soil porosity and hydraulic conductivity and increases soil-bearing capacity and shear resistance. MICP therefore offers applications within the civil and earthquake engineering sectors (DeJong et al., 2013; Montoya et al., 2013).

MICP is considered to be “environmentally friendly” compared to traditional soil stabilisation techniques, although this statement needs clarity. Firstly, although indigenous soil bacteria are reported equally suitable (Burbank et al., 2010, 2012, 2013), exogenous bacterial communities grown in the laboratory have been typically used for MICP which suffer predation by indigenous bacterial communities once introduced into the soil environment. Secondly, principal chemical components required for MICP such as organic C and N sources (urea, sodium acetate, molasses, yeast and ammonium chloride) and CaCl_2_ are industry-end products. Thirdly, there is a paucity of information concerning the potential for contamination of aquifers through post-treatment nitrification processes and the soil-atmosphere Greenhouse Gas (GHG) emissions. CO_2_ sequestration during MICP by certain ureolytic communities has been observed (Okyay et al., 2015, 2016). However, these studies were conducted in an aquatic environment at atmospheric CO_2_ levels of 10%, which differ from soil environments.

With a global movement towards a circular economy, alternative sources to CaCl_2_ have been suggested including seawater (Cheng et al., 2014), eggshells (Choi et al., 2016), limestone (Choi et al., 2017), and basic silicate rocks (Casas et al., 2019). Dolerite is a rock of basaltic chemical composition abundant in Ca-rich minerals, which releases Ca and Mg in solution during chemical weathering. Dissolution of basaltic rocks is associated with atmospheric CO_2_ removal via the formation of carbonate minerals occurring at the mineral surface and underlaying soil layers by downward percolation of divalent cation-rich alkaline solution (Manning et al., 2013). Dolerite fines of large specific surface area originate from crushing and milling processes in quarries and often accumulate in landfill due to limited market options (Mitchell et al., 2008). This material could, however, be suitable for MICP and CO_2_ capture applications (Casas et al., 2019). Furthermore, induced toxicity by dolerite weathering has not been reported up to now.

Enhanced weathering of basic silicate rocks has been previously reported for the potential mitigation of anthropogenic CO_2_ emissions (Manning, 2008; Renforth et al., 2009; Renforth, 2012). The need for evaluating CO_2_ capture strategies, such as the application of basic silicate rocks to soil, combined with detailed environmental monitoring, has been discussed (Beerling et al., 2018). In this context, this paper reports investigations of soil-atmosphere CO_2_ fluxes during MICP treatment of a natural sandy soil using CaCl_2_ and dolerite fines originating from quarries to determine stages where the system may act as a source or sink of CO_2_ and to identify the main drivers of these fluxes. Furthermore, the CO_2_ sequestration potential of dolerite fines by chemical and biological weathering were investigated.

## Materials and Methods

### Soil Sampling and Characterisation

Sand was obtained from a quarry located in the Lower Rhine Basin, Germany (50°55′18.8184″, 06°46’45.6528″, WGS84; operated by Quarzwerke GmbH, Frechen). The sampling location was selected close to vegetation, thus ensuring that the soil was biologically active. The surface 3 cm were removed and the underlying soil to a depth of 20 cm collected and sealed to prevent moisture loss. The sample consisted of a loose, fairly homogeneous white-greyish fine sand, with no presence of gravels, boulders or fines. Thin plant roots were present. The soil moisture content was determined gravimetrically upon arrival at the laboratory. The grain size distribution was determined by wet sieving on two analytical replicates (ISO11277), and the soil pH was determined in 0.1 M CaCl_2_ solution for a soil-to-solution ratio of 2.5:1 on three analytical replicates (WTW SenTix 41 PLUS probe with WTW multi 340i pH meter, WTW, Weilheim, Germany; calibrated to pH = 4 and 7).

### Dolerite Fines

Dolerite fines were sourced from Barrasford quarry (Barrasford, Hexham, NE48 4AP, United Kingdom; operated by Tarmac Ltd.). The chemical composition and mineralogy of the quarry materials are described in detail in Randall (1989) and the physical properties, chemical composition, and calcium chemical weatherability of the material discussed previously by Casas et al. (2019).

### MICP Treatment Solutions

MICP was induced through biostimulation of soil indigenous bacteria. The chemical composition of treatment solutions was defined according to Burbank et al. (2010) and Al Qabany and Soga (2013). The growth solution contained 1 g L^–1^ of cane molasses (MLS) (Rapunzel Naturkost GmbH, Germany), 0.1 g L^–1^ yeast extract (Vitasan Bio-Hefeextrakt; VITAM Hefe-Produkt GmbH, Germany), 100 mM anhydrous sodium acetate (SA) (ACS, Merck KGaA, Germany) and 250 mM urea (≥99.5%, Carl Roth GmbH + Co., KG, Germany) in distilled water. The cementation solution contained 0.1 g L^–1^ of MLS, 100 mM SA, 100 mM urea and either 0 or 20 mM CaCl_2_ ⋅ 2 H_2_O (ACS, Merck KGaA, Germany) in distilled water. The MICP treatment sequence consisted of several phases and subphases detailed in section “MICP and the Application of Dolerite Fines” and Supplementary Figure S2.

### CO_2_ Measurements

#### Dissolution of Dolerite Fines

The CO_2_ sequestration potential of dolerite fines at low liquid-to-solid (L/S) ratios was investigated by gas chromatography (GC). Samples were prepared by adding a constant mass of dolerite fines (0.1 g) and varying amounts of distilled water in 22 mL GC vials, sealed using butyl rubber septa and aluminium caps, manually stirred, and incubated. The evolution of CO_2_ concentration during dolerite dissolution by chemical weathering over a 1 h incubation period was measured after 5, 20, 40, and 60 mins, for L/S ratios of 0, 0.6, 1.0, 1.5, 2.5, 5, 10, and 15. The experiments were repeated every 24 h. At the end of each experiment, vials were opened and stored at room temperature. Distilled water equivalent to the evaporated water was then added right before closing the vial to maintain a constant L/S ratio. All experiments were carried out in triplicate. CO_2_ concentrations were determined by gas chromatography (GC 8610C, SRI Instruments Europe GmbH, Bad Honnef, Germany) interfaced to an HTA-40 autosampler equipped with a packed pre-column (1 m Porapak-Q 1/8″) and a flame ionisation detector (FID) using nitrogen as the carrier gas. Four gas measurements were made on the same vial during a single incubation. The GC sampled 3.5 mL of headspace gas per measurement without control of the vial inner pressure. A pressure drop of 0.2 bar per measurement was determined in a preliminary study. Corrections for this were carried out using an algorithm using python (script available upon request). Vials were brought to 1.3 bar above ambient air pressure with N_2_ before the first measurement to maintain overpressure conditions within the vial after four measurements. The data was fitted to equation:

(1) $$C\,\left(t\right)=C\,\left(x\right)+\left(C_{0}-C_{x}\right)\,e^{-a\,\left(t-t_{0}\right)}$$

where *C(t)* is the CO_2_ concentration at time *t*, *C*_0_, and *C*_x_ correspond to the CO_2_ concentration at *t*_0_ and at equilibrium, respectively, and *a* is the exponential constant determined for each L/S ratio.

#### MICP and the Application of Dolerite Fines

CO_2_ fluxes between soil and atmosphere during MICP were investigated in a soil column setup coupled to a soil CO_2_ efflux monitoring chamber system (LI-8100, LI-COR Biosciences, LI-COR Inc., United States) (Supplementary Figure S1). In a replicate soil column, the effect of a layer of dolerite fines placed on top of the soil surface to be treated with MICP was investigated. The function of the dolerite layer was to release calcium into the soil column when reacting with the poured solution via chemical weathering. This alternative source of calcium was compared against the MICP solution containing industrially manufactured CaCl_2_. Soil samples were prepared in each column by adding 3 kg of soil, resulting in a soil layer of 8.5 cm. 500 g of dry dolerite fines were placed on top of one of the soil columns resulting in a 1 cm layer. Dolerite fines were applied after the growth phase to maximise calcium availability for precipitation. Top to bottom, the soil column setup consisted of a soil chamber (*A* = 314.16 cm^2^) resting on top of a cylindrical PVC tube (11.5 cm height and 20 cm i.d.), with a sieve (0.036 mm mesh) fitted at the bottom, joined to a drainage system composed of a metallic receiver attached to a plastic tube (4 mm i.d.) and a valve. Silicone was used to join and seal the assembled parts.

The MICP treatment consisted of a 4-day growth followed by an 8-day cementation phase. A month after finalising the treatment soil columns were rewetted with distilled water. Solutions were applied by gravitational drainage (percolation) controlled by the outlet valve mechanism. Treatments comprised a reaction time (*t*_r_) and rest time interval. During the reaction time the outlet valve was closed to keep soil and solution in contact, and the soil and dolerite surfaces remained submerged. Rest intervals comprised the time between opening the valve for solution drainage and addition of fresh solution. A drying phase was induced when solution drainage was not followed by another treatment consecutively. The reaction time of the growth phase was 96 h. The cementation phase commenced 24 h later and consisted of several individual treatments of 24 h duration, with reaction and rest time intervals of 23 and 1 h, respectively. In total, eight cementation treatments were applied, split in two rounds of four consecutive treatments with a 72-h drying phase in between. For each treatment, a total of 1 l was poured in each soil column, only retaining 500 mL to maximise solution replacement within the soil column. The MICP treatment phase was followed by a drying phase of 28 d. The soil columns were then flushed with 1 l of distilled water, retained for 24 h, and ensued by a third drying phase of 21 d.

CO_2_ measurements were carried out throughout the MICP treatment and the drying phases that followed, covering a total timespan of 2 months. The chambers of the CO_2_ efflux monitoring system were open to allow free gas exchange between the soil surface and the atmosphere and closed every full hour for 10 min for a measurement. During this time, CO_2_ concentration, H_2_O vapor concentration and temperature inside the chamber were logged at a frequency of 1 s^–1^, and CO_2_ efflux or uptake of the sample was computed from the initial slope of a non-linear curve fit to the increase or decrease of humidity-corrected CO_2_ concentrations. Liquid and solid samples were obtained after each cementation treatment, as well as after re-wetting the soil columns. Liquid samples were tested for pH upon sampling and stored at −20°C until further analysis (see section “Chemical Analyses of Liquid Samples”). Soil samples were obtained pushing down a cylindrical glass tube (4.5 mm i.d.) into the soil profile, oven-dried and analysed for soil total inorganic carbon (TIC) and isotopic composition (see section “Quantitative and Isotopic Analysis of C”).

### Chemical Analyses of Liquid Samples

The chemical composition of soil leachates was determined after the first and last cementation treatments, and after rewetting of soil columns. pH, total organic carbon (TOC), total nitrogen (TN), urea, ammonium, nitrite, nitrate and elements Ca, Na, Mg, K, Mn, Fe, Al, Ti, Si, B, Ba, Cd, Co, Cr, Cu, Mo, Ni, P, S, Se, Sr, V, W, and Zn in solution were determined. Liquid samples were centrifuged for 10 min and the supernatant analysed for TOC and TN (TOC-L Analyser with TNM-L unit, Shimadzu Ltd., United Kingdom) and elements determined by inductively coupled plasma optical emission spectroscopy (IC-OES, Perkin Elmer Avio 500) (James Hutton Ltd., Craigiebuckler, Aberdeen, United Kingdom). For ICP-OES analysis, samples obtained after rewetting of soil columns were pre-treated with a hot acid digest as the presence of amorphous calcium carbonate (ACC) was suspected, all other samples were diluted into a nitric acid matrix. Urea [*r*^2^ = 0.999, SD_max_ = 0.04 mM; Knorst et al. (1997)], ammonium, nitrate and nitrite (cuvette tests LCK303, LCK339 and LCK341, Hach Lange GmbH) were determined colourimetrically in triplicate by spectrophotometer (DR 5000^TM^ UV-Vis Spectrophotometer, United States).

### Quantitative and Isotopic Analysis of C

Soil TOC, TN and the C isotopic signature (δ^13^C_TOC_) were determined in triplicate by elemental analysis–isotope-ratio mass spectrometry (EA-IRMS) using an elemental analyser (EA; Flash 2000, Thermo Fisher Scientific^TM^) interfaced with a continuous flow IRMS (CF-IRMS; Delta V Advantage, Thermo Fisher Scientific, Bremen, Germany). Soil TIC and the isotopic composition of C (δ^13^C_TIC_) and O (δ^18^O) were determined by gas chromatography–continuous flow–isotope ratio mass spectrometry (GC-CF-IRMS, GasBench II interfaced with a Delta V Advantage, Thermo Fisher Scientific). Soil TOC and TIC were determined in separate runs. For analysis of TOC, TIC was previously removed by fumigation of pre-weighed samples with concentrated hydrochloric acid vapour (Ramnarine et al., 2011). TIC was determined by analysis of evolved CO_2_ gas released by the reaction of carbonate and pure phosphoric acid (Debajyoti and Skrzypek, 2007). The CO_2_ content evolving in the vial headspace (12 mL screw top exetainers sealed with septa) was estimated by comparing peak areas of samples against air samples using GC and IRMS. The isotopic composition of C-containing compounds used for the preparation of growth and cementation solutions (i.e., molasses, urea and sodium acetate) was determined for each individual compound by EA-IRMS. Certified isotopic standards were used for ^13^C, referenced against the Vienna Pee Dee Belemnite (VPDB).

## Results

### Material Characterisation

The soil used in this study was classified as poorly graded sand (SP, USCS unified soil classification system) with 29% medium, 67% fine sand, and fines content <4% (Table 1). The moisture content was very low at θ≈ 1%. Soil organic C (C_TOC_ = 0.0211 wt%) and carbonate (C_TIC_ = 0.0003 wt%) content were also low, the C:N ratio was 13 and the soil pH was slightly acidic (pH = 6.4). The isotopic signature of soil organic and inorganic C was δ^13^C_TIC_ = −14‰ and δ^13^C_TOC_ = −26‰, respectively (Table 1). The C-containing compounds used for the preparation of the growth and cementation solutions had a similar organic carbon content (C_org_ = 20–32%) while the C_org_ isotopic signature differed (δ^13^C_urea_ = −40‰, δ^13^C_SA_ = −31‰ and δ^13^C_MLS_ = −13‰) (Table 2).

**TABLE 1:** Soil physical and chemical properties.

Parameter	Unit	Value
Sand	wt%	96.2
Fines	wt%	3.8
D_60_	mm	0.18
D_30_	mm	0.1
D_10_	mm	0.07
C_u_ = D_60_/D_30_	−	2.57
C_c_ = (D_30_)^2^/(D_60_ D_10_)	−	0.79
pH	−	6.4
TIC	wt%	0.0003
TOC	wt%	0.0211
TN	wt%	0.0017
C/N	−	12.9
δ^13^C_TIC_	‰, relative to VPDB	−13.9
δ^13^C_TOC_	‰, relative to VPDB	−25.7

**Particle size distribution (n = 2), pH (n = 3), TIC and δ^13^C_TIC_ (n = 2), TOC and δ1^3^C_TOC_ (n = 4, treated and untreated), TN (n = 2). SD for TIC, TOC and TN < 0.0006 and δ^13^C < 1.8‰.**

**TABLE 2:** Total organic carbon and isotopic signature of organic carbon in sodium acetate, urea, and molasses.

Chemical compound	TOC (wt%)	δ^13^C_org._ (‰, relative to VPDB)
Sodium Acetate (SA)	28.6 ± 0.3	−30.49 ± 0.27
Molasses (MLS)	31.9 ± 0.2	−12.58 ± 0.07
Urea	20.1 ± 0.2	−41.03 ± 0.12

**Replicates (n) for sodium acetate, n = 3; molasses, n = 3 and urea, n = 6. Standard deviation indicated after “±”.**

### CO_2_ Fluxes During MICP

**FIGURE 1:**
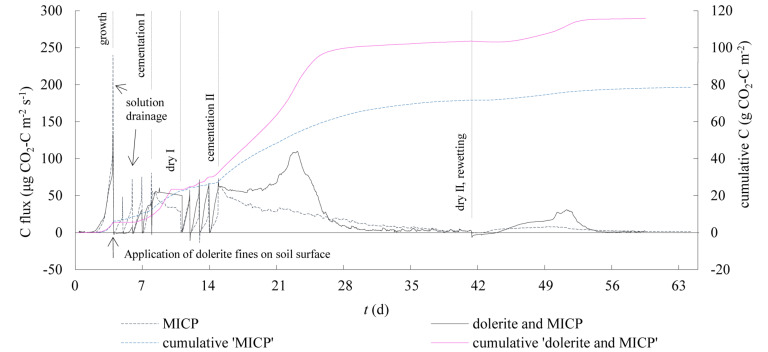
Time series of soil-atmosphere CO_2_ fluxes of sandy soil columns treated with the traditional MICP treatment (MICP) and using dolerite fines applied on the soil surface as source for calcium (dolerite and MICP). CO_2_ fluxes and cumulative CO_2_ emission link to left and right vertical axes, relatively. Divisions on bottom horizontal axis represent 1 d. The application of dolerite is indicated with a vertical arrow and ‘solution drainage’ are pointed as examples. The end of the experiment phases are indicated with dropping vertical dashed lines.

Positive CO_2_ fluxes indicated emission dominated over the 2-month period comprising MICP treatment and drying phases (Figure 1). CO_2_ emissions steadily increased during reaction time intervals following linear trends. Sharp CO_2_ emission peaks were measured at the points of solution drainage. Following drainage, CO_2_ fluxes fell to near zero if a new treatment was applied but remained high when a drying phase ensued. For the column treated with the traditional MICP solution, CO_2_ emissions decreased steadily approaching zero following the MICP treatment. Instead, from the column containing dolerite fines, emissions remained steady for a week, followed by increased emissions with a peak occurring 8 d following the beginning of the drying phase prior to decay in CO_2_ emissions (Figure 1). This pattern was repeated in smaller proportion after rewetting the soil column.

**FIGURE 2:**
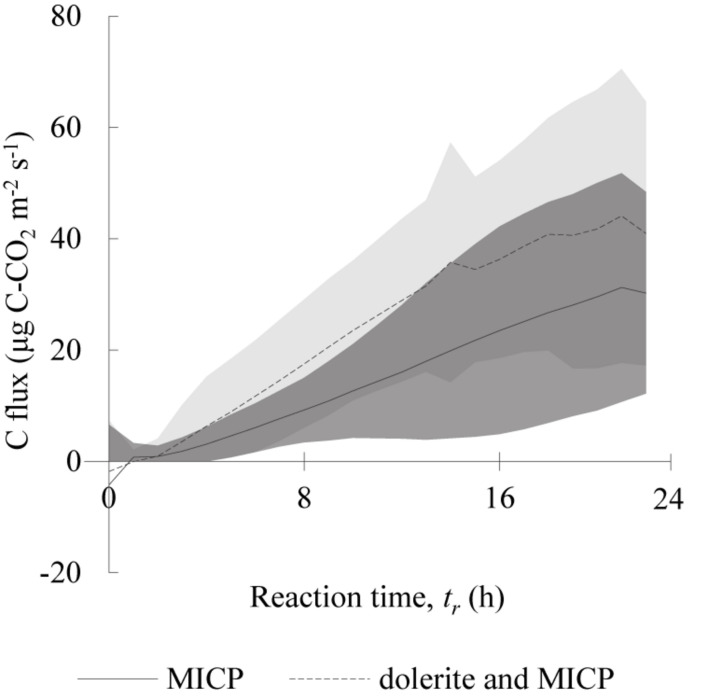
Average CO_2_ fluxes during 24 h reaction time of soil column treated with traditional (MICP), and for soil column containing dolerite fines added to the MICP treatment (dolerite and MICP). The first and second treatments were excluded from the computed average for both treatments for comparison purposes and to remove the initial near-zero CO_2_ fluxes not observed in posterior treatments in the soil column containing dolerite fines. Dark and light grey shaded areas indicate ± two standard deviations for MICP and dolerite and MICP, respectively.

Total emissions were larger for the soil column containing dolerite fines (116 g CO_2_-C m^–2^) compared to the soil column treated with the traditional MICP solution (79 g CO_2_-C m^–2^). In both cases, most emissions occurred during the drying phases (78–84%), with more than half (55–60%) occurring during the drying phase following the end of the MICP treatment. Notably, near-zero CO_2_ fluxes in the soil column containing dolerite fines were monitored during the initial two cementation treatments but there were higher CO_2_ emissions from the third cementation treatment onwards. Cumulative CO_2_ fluxes indicated overall CO_2_ emissions were lower for up to 6 days following application of dolerite fines on the soil surface compared to the traditional MICP treatment (Figure 1). The average and standard deviation of discrete CO_2_ fluxes of a 24 h cementation treatment are presented in Figure 2, were the initial two treatments were removed for comparison purposes. According to the computed averages, 25% of CO_2_ emissions during reaction time were emitted in the initial 6 and 8 h and 50% in the 10–12 h, for the column treated with traditional MICP and the MICP with dolerite fines treatment, respectively. The computed average soil respiration of a 24 h cementation treatment were of 1.33 ± 0.44 and 2.01 ± 0.55 g CO_2_-C m^2^ for the traditional and the MICP treatment with dolerite fines, respectively.

### Chemical Analyses on Soil Leachates

**FIGURE 3:**
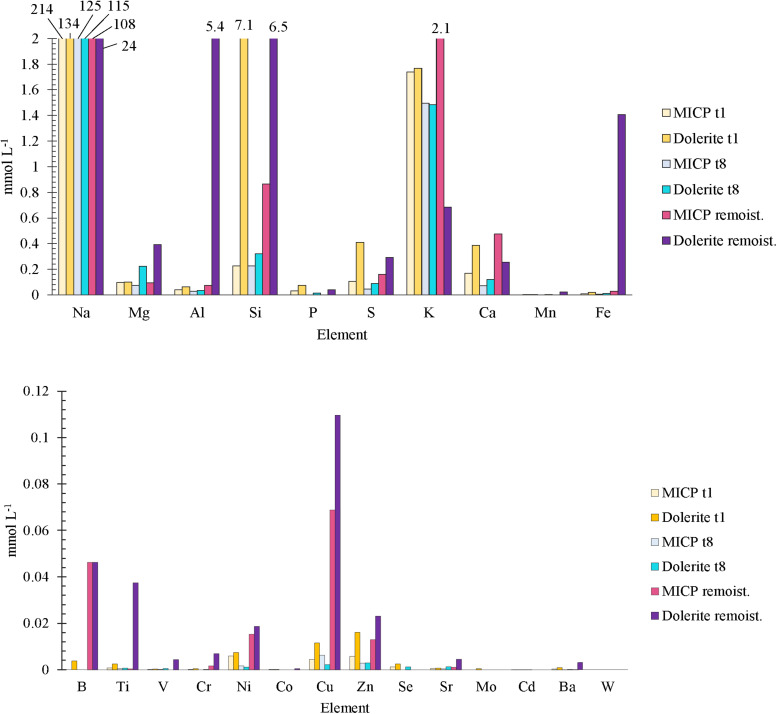
ICP-OES analysis (*n* = 1) of common **(above)** and trace **(below)** weathered elements dissolved in soil leachate samples obtained after first (*t*_1_) and last (*t*_8_) cementation treatments and a month after finali**s**ing the treatment (rewetting) for columns treated with traditional MICP and soil column containing dolerite fines treated with MICP (Dolerite). Analysis carried out by James Hutton Ltd., Aberdeen, United Kingdom.

Soil leachates had a pH above 9 indicating urea hydrolysis and favourable environmental conditions for the formation of carbonate minerals (data not shown). ICP-OES analysis indicated Ca (<0.5 mM) was removed from the soil aqueous phase (Figure 3A). Ca, Mg, S, P, Si, and Al, present in both soil leachates at <1 mM, were found in larger concentration in the leachate obtained from the column containing dolerite fines. Si concentration was particularly high (7.8 mM) after the first cementation treatment. A month after finalising the treatment, the soil leachate from the column containing dolerite fines contained Si (6.3 mM), Al (5.4 mM), and Fe (1.4 mM), while for the column treated with the traditional MICP solution these elements remained <1 mM. Further, low concentrations (<0.1 mM) of Cu, Ti, Zn, Ni, Mn and Ba were detected (Figure 3B). Elemental analysis of TN and colorimetric analysis of urea, ammonium, nitrite and nitrate indicated that N, mainly ammonium (>66%), decreased to near-zero values during the drying phase that followed the end of MICP treatment until soil columns were rewetted. Urea hydrolysis rates between 2.038 and 4.374 mM h^–1^ were determined during the MICP cementation phase. Nitrite (<0.05 mM) and nitrate (<3 mM) were low throughout (Supplementary Table S2).

### Soil Inorganic Carbon Content and Isotope Analysis

**FIGURE 4:**
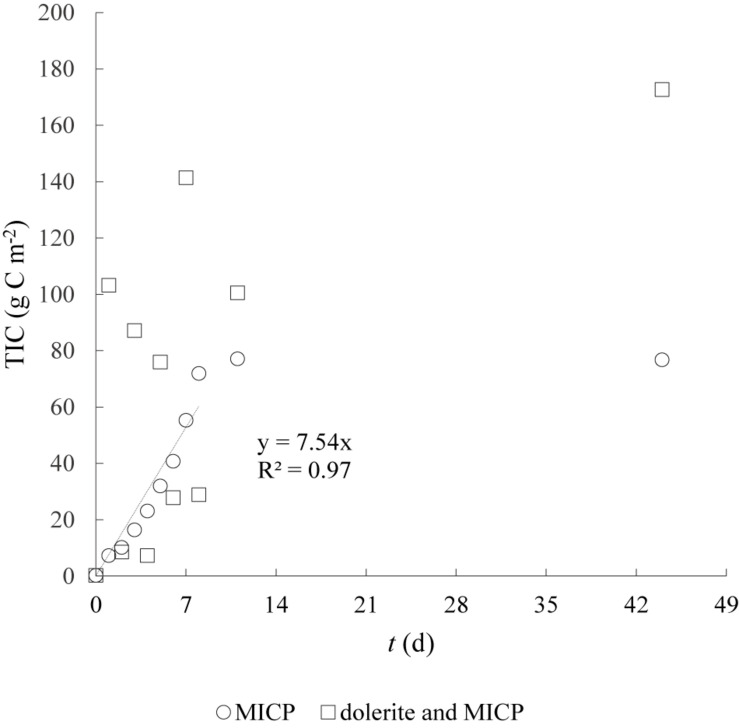
Soil total inorganic carbon during the MICP treatment phase for soil column treated with traditional MICP solution (MICP) and soil column containing dolerite fines (dolerite and MICP) determined by EA-IRMS (*n* = 2; *SD* < 0.002, SD smaller than markers). Regression line and equation fit of traditional MICP treatment data.

An increase in soil inorganic carbon (TIC) was observed during the treatment phase compared to the untreated soil inorganic C content (Figure 4). This indicated that carbonate minerals formed within the soil matrix in both treatments. For the soil column treated with the traditional MICP solution, TIC content increased linearly (*r*^2^ = 0.97) with the number of cementation treatments up to 77.16 g C m^–2^, with an average TIC accumulation of 7.54 g C m^–2^ per treatment. A month after finalising the treatment no further change in TIC was detected. For the soil column treated with dolerite fines, TIC (7.2–141.5 g C m^–2^) content was generally larger compared to the traditional MICP treatment. A month after finalising the treatment, the TIC content had increased further to 172.8 g C m^–2^. Combining these differences in precipitated C with the emissions reported in section “CO_2_ Fluxes During MICP,” the environmental cost of C emitted as CO_2_ per C precipitated was larger for traditional MICP (1.01) than for the dolerite fines treatment (0.67).

**FIGURE 5:**
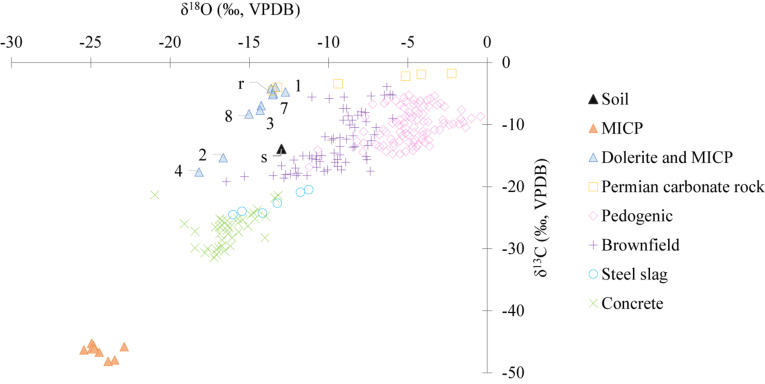
Stable isotopic signature of precipitated carbonates (δ^18^O ‰ and δ^13^C ‰, VPDB) (*n* = 2; *SD* < 1.9, SD smaller than markers) in soil column treated with traditional MICP (MICP) and soil column containing dolerite fines treated with MICP (dolerite and MICP). Numeric labels indicate the treatment number and “r” label stands for “re-wetting.” Data from concrete (Macleod et al., 1991; Krishnamurthy et al., 2003), steel slag (Renforth et al., 2009), brownfield sites (Washbourne et al., 2015; Jorat et al., 2020), Permian carbonate rock (Harwood and Coleman, 1981), and pedogenic soil carbonates (Salomons et al., 1978) included for comparison.

The results of the C and O isotopic analysis of precipitated carbonates differed markedly between the two treatments (Figure 5), with average values of δ^18^O = −24.1‰ and δ^13^C = −46.4‰ relative to VPDB for the column treated with the traditional MICP solution, and δ^18^O = −14.6‰ and δ^13^C = −8.2‰ for the column treated with dolerite fines and MICP. The isotopic signature of TIC precipitated after treatment with the traditional MICP solution was similar to that of C in urea (δ^13^C = −41.03‰). In contrast, for the column containing dolerite fines, δ^13^C values were similar to geological and pedogenic C, although δ^18^O values were more negative (Figure 5). There was a strong linear correlation (*r*^2^ = 0.95) between δ^18^O and δ^13^C values with a slope of 2.78 and an intercept of 32.4 for the column containing dolerite fines. In contrast, values of the column treated with the traditional MICP solution formed a cluster without clear linear relationship.

### CO_2_ Gas Measurements During Dolerite Fines Dissolution

**FIGURE 6:**
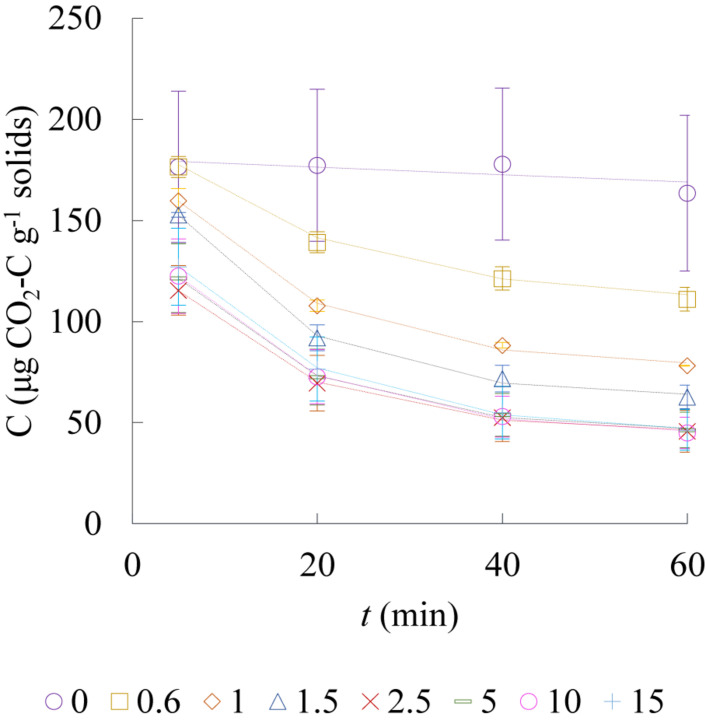
Evolution of CO_2_-C normalised to the mass of solids in vial headspace during chemical weathering of dolerite fines in distilled water at fixed liquid-to-solid ratios (*L*/*S* = 0, 0.6, 1.0, 1.5, 2.5, 5, 10, and 15) over 60 min, analysed with gas chromatography (*n* = 3; SD indicated with error bars). Lines correspond to the fitting model described by Eq. (1). Data corresponds to experiment day 1.

The CO_2_ sequestration potential of dolerite fines during chemical weathering at low liquid-to-solid ratios was studied in an isolated environment by gas chromatography. Results fitted with Eq. (1) indicated that the concentration at the equilibrium boundary value decreased from almost 170 to around 46 μg CO_2_-C per g of solids with increasing water content (Figure 6). Repeated water additions on consecutive days led to smaller CO_2_ variation in the vial headspace such that the exponential fit was found unsuitable due to the flat trends (data not shown). The reaction of CO_2_ with water and dolerite fines was accelerated with increasing water content up to a L/S ratio of 1.5 and levelled off for higher L/S. Increasing the liquid to solid ratio above 2.5 and up to 15 did not lead to larger CO_2_ sequestration (Figure 6) but reduced significantly the capacity of the material to sequester CO_2_ on consecutive water additions via chemical weathering (data not shown).

## Discussion

Our study on soil-atmosphere CO_2_ fluxes of a sandy soil treated with MICP showed net CO_2_ emissions throughout the treatment phase and the following 3 weeks of post-treatment. The reaction time of a cementation treatment and the soil water saturation conditions played an important role in the observed total CO_2_ emissions, with emissions increasing with time during reaction time intervals and decreasing with the addition of fresh solution. In partially saturated conditions, occurring during the drying phases, emission levels remained high with larger CO_2_ emissions resulting from partially saturated conditions compared to submerged conditions, similar to observations reported by Rahman (2013). The bacterial activity decayed following the traditional MICP treatment, as indicated by the decreasing CO_2_ emissions (Figure 1). The linear increase in soil TIC during the MICP treatment indicated that the formation of calcium carbonate was limited by the availability of Ca and, following treatment, TIC built up in soil (Figure 4) was limited by the depletion of divalent cations. The isotopic analysis showed the precipitated carbonate C (−46‰) originating from urea is similar to reported δ^13^C values of carbonate C precipitated during MICP (Millo et al., 2012).

The low contents of TOC, TIC, fines and low soil moisture indicated that the soil was mainly composed of pure silica sand, and the isotopic signature of TOC (−26‰) and TIC (−14‰) indicated the soil C to be of C_3_ plant biogenic origin (Cerling, 1984; Manning, 2008). Thus, in the absence of organic matter in decomposition, the differences in CO_2_ emission trends could be attributable to differences in soil microbial response to the environmental conditions created by the presence or absence of dolerite fines. Applying dolerite fines to the soil surface led to larger CO_2_ emissions and larger precipitation of inorganic C compared to the traditional MICP treatment. CO_2_ emissions during the reaction time were found to be on average two times larger than for the common MICP solution treatment (Figure 2). Based on the average values presented in Figure 2, CO_2_ emissions of a typical MICP cementation treatment could be reduced by 75% with reaction time shorter than 8 h and by 50% with reaction time shorter than 10 h. While urea hydrolysis rates may vary greatly for different soil types and whether MICP is induced by stimulation of indigenous bacteria or by bioaugmentation (introduction of exogenous ureolytic bacterial strains), results on clean silica sand presented by Al Qabany and Soga (2013) indicated 8 h of reaction time were sufficient for optimum degradation of urea and production of carbonates. The larger CO_2_ emissions observed after treatment pointed to an enhanced biological activity, fuelled by the presence of oxygen and the availability of elements released by biological weathering of dolerite fines. Dolerite fines dissolution in the biologically active environment balanced CO_2_ emissions induced by the treatment for approximately 1.5 d after application (Figure 1). The pH of outlet liquid samples of both columns was above 9, indicating urea hydrolysis occurred and resulted in CO_2_ emissions in the column that did not contain dolerite fines. Instead, near-zero CO_2_ fluxes were monitored for the soil column containing dolerite fines and a large increase in precipitated inorganic carbon was also recorded (Figure 4). This was also observed during chemical weathering experiments, where the large CO_2_ sequestration capacity of the material decreased dramatically after 24 h. The chemical weathering experiments showed that CO_2_ sequestration rates increased with increasing water content up to L/S ratios of 1.5–2.5, while larger L/S ratios accelerated the drop in CO_2_ sequestration capacity resulting in flat trends on the second consecutive day the experiment was run with the same material (data not shown). Calcium carbonate precipitated on silicate mineral surfaces was not found to decrease dissolution kinetics of diopside by Stockmann et al. (2013) suggesting that the weatherability and the CO_2_ sequestration capacity of dolerite fines were initially controlled by the reactive mineral surface area. Far-from-equilibrium conditions accelerated reactive surface area reduction, therefore decreasing the large short-term CO_2_ sequestration capacity of the material.

Our data supports biological weathering played significant role in overall rock weathering and TIC accumulation, as dolerite fines showed a large but short-lived chemical weathering capacity and the environmental conditions produced by urea hydrolysis were highly alkaline. The large initial Si release and Ca, Mg and Al determined in soil leachates (Figure 3) indicated a continuous release of elements by dolerite fines during the MICP treatment. CO_2_ emissions and the large concentration of Al, Si and Fe and of Ca, Mg, P, S and other elements indicated rock weathering and soil biological activity continued throughout the drying phase following the MICP treatment. The Fe was possibly in its oxidised state and thus unable to form carbonates, given the availability of inorganic C and alkaline pH. The application of dolerite fines was not found to significantly enhance nor inhibit the activity of ureolytic microorganisms, as indicated by the similar urea hydrolysis rates, pH, and ammonia levels determined in soil leachates of both treatments. Our data indicate that urea was completely hydrolysed 24 h after application, supporting previous studies (e.g., Haynes and Williams, 1992). Petersen et al. (2004) showed that urea-C, applied at concentrations similar to those in this study, contributed less than 1% of the total soil CO_2_ emissions 4 days after application. This and the larger CO_2_ emissions during reaction time of the cementation treatment (Figure 2) indicated that the larger CO_2_ emissions observed for the column containing dolerite fines may not be solely attributable to the activity of ureolytic microorganisms, but to an overall increased soil respiration. The increased CO_2_ emissions detected between 6 and 12 days after finalising the MICP treatment may be indicative of favourable environmental conditions, provided by moisture and ammonia removal from the environment by evaporation, volatilisation and nitrification. However, nitrous oxides were determined at low concentrations, and nitrification may have been inhibited as a result of ammonia toxicity (Anthonisen et al., 1976; Smith et al., 1997; Petersen et al., 2004). Whether the availability of elements provided by the treatment and the presence of dolerite was the cause of diversification of stimulated soil microbiota and/or allowed for larger community numbers and was responsible for the larger CO_2_ emissions observed should be investigated further.

The large variability observed in TIC did not necessarily correspond to soil TIC, but to TIC precipitated on dolerite and soil. The isotopic signature of carbonate C (−8.2‰), similar to carbonates from Permian carbonate rocks (Harwood and Coleman, 1981), pedogenic carbonates formed in soils (Salomons et al., 1978) and brownfield sites (Washbourne et al., 2015; Jorat et al., 2020) and steel slag carbonates (Renforth et al., 2009) indicated the carbonate C was not derived from urea-C. Instead, the data indicated that carbonates formed as a combination of “natural” pedogenic carbonate formation processes combined with carbonates formed in highly alkaline environments by hydroxylation of dissolved CO_2_, and that both mechanisms were controlled by CO_2_ gas diffusion in solution (Dietzel et al., 1992; Renforth et al., 2009). This suggested that weathered Ca readily reacted with dissolved inorganic C within the dolerite layer and precipitated on silicate mineral surfaces, while unattached carbonates percolated through the soil column. This may have been additionally favoured by the alkaline conditions induced by urea hydrolysis. While TIC in soil treated with dolerite and MICP was generally higher than in the traditional MICP treatment, slightly lower TIC values observed for some samples in Figure 4 compared to the traditional MICP treatment and may indicate that carbonate precipitation occurred to a lesser extent within the soil layer. However, TIC analysis were not conducted at different depths nor a specific procedure to ensure separation of the different materials (soil and dolerite) was conducted. Additionally, while dolerite was sampled along with soil in some cases, as presented in Supplementary Figure S3 as example, this was not always the case. Therefore, the observed variability in precipitated TIC may be due to several factors. Variability of TIC analysis could be due to non-homogeneity of precipitated TIC produced by the low permeability of dolerite fines (Casas et al., 2019) compared to the soil layer, which could have created preferential paths, resulting in TIC building up in some areas more than others. Similarly, samples taken for TIC analysis could contain variable fractions of dolerite fines that percolated through the soil matrix in some areas more than others. Geotechnical tests were not conducted on soil columns after the treatment, therefore, TIC data suggests that these aspects should be further investigated to elucidate the suitability of dolerite fines for certain applications, such as for soil stabilisation.

## Conclusion

Our study on CO_2_ fluxes during MICP on sandy soil using CaCl_2_ and dolerite fines as sources for calcium demonstrated large CO_2_ emission levels are the result of microbial activity induced by the treatment. The emissions were not balanced by the removal mechanism through carbonate precipitation due to the limited availability of divalent cations. The post-treatment biological activity was enhanced by the presence of dolerite fines and resulted in larger CO_2_ emissions and increased overall TIC content. However, from the present experiments it remains unclear how much TIC formation took place in the soil itself. Biological weathering of dolerite fines was revealed by the presence of Al and Si in the soil leachate and indicated the potential for long-term TIC accumulation. CO_2_ emissions of MICP treatment were reduced with dolerite fines and isotope data showed atmospheric CO_2_ was sequestered in the presence of dolerite whereas traditional MICP primarily sequestered C from urea. Overall CO_2_ emissions could be reduced through optimising the flushing-reaction time sequence. Maintaining submerged conditions and reaction times within 8–10 h could reduce CO_2_ emissions of a typical MICP treatment by 50–75%. However, reaction time should be adapted to specific soil urea hydrolysis rates to ensure sufficient production of carbonate. Further, CO_2_ emissions could be directly balanced via chemical weathering by the application of unweathered dolerite fines due to the large reactive surface area of the material. In this aspect, maintaining liquid to solid ratios equal or below 2.5 would maximise the capacity of dolerite to balance CO_2_ emissions. We postulate that these results are applicable to other fields of research, such as agriculture. The soil environmental conditions induced by the MICP process resemble those generated with the application of natural and synthesised N and Ca-containing fertilisers on land. The effect of soil organic matter and living plants should therefore be incorporated in further studies.

## Data Availability Statement

The datasets generated for this study are available upon request.

## Author Contributions

MEJ and CS were project supervisors. MEJ, AG, NB, and CC contributed to the experimental design. AG and CC carried out the experiments. AG, NB, and CC contributed to the analysis of data. CC wrote the original draft manuscript. MEJ, AG, NB, and CS reviewed and edited the manuscript. All authors contributed to the article and approved the submitted version.

## Conflict of Interest

The authors declare that the research was conducted in the absence of any commercial or financial relationships that could be construed as a potential conflict of interest.

## Abbreviations

C: Carbon
CO_2_: Carbon dioxide
DIC: Dissolved inorganic carbon
N: Nitrogen
O: Oxygen
TIC: Total inorganic carbon
TN: Total nitrogen
TOC: Total organic carbon.
