# Supplementary material for: Dolerite Fines Used as a Calcium Source for Microbially Induced Calcite Precipitation Reduce the Environmental Carbon Cost in Sandy Soil
Source: Front Microbiol. 2020 Sep 8;11:557119. doi: 10.3389/fmicb.2020.557119 (PMC7505998; doi:10.3389/fmicb.2020.557119)
Supplement: Supplementary file 1 [file Data_Sheet_1.docx]

Supplementary Material

# Table of contents

[Table of contents 1](#_Toc38441604)

[List of figures 1](#_Toc38441605)

[List of tables 1](#_Toc38441606)

[1 Methods 2](#_Toc38441607)

[1.1 Soil column setup 2](#_Toc38441608)

[1.2 Soil column experiment phases 3](#_Toc38441609)

[1.3 Soil sampling 3](#_Toc38441610)

[2 Results 4](#_Toc38441611)

[2.1 ICP-OES analysis of soil leachates 4](#_Toc38441612)

[2.2 Elemental C and N analysis and N compounds in soil leachates 5](#_Toc38441613)

# List of figures

[Figure S1 Soil column setup and measuring equipment 2](#_Toc38441139)

[Figure S2 Scheme of the experiment phases over the two months period. 3](#_Toc38441140)

[Figure S3 Soil sampling detail showing samples obtained for soil analysis and dolerite fines from top layer were sampled with soil 3](#_Toc38441141)

# List of tables

[Table S1 Elements ICP-OES (n =1). “t” stands for treatment. Analysis carried out by James Hutton Ltd., Aberdeen, UK 4](#_Toc38441142)

[Table S2 TOC, TN, urea-N, ammonium-N, nitrite-N and nitrate-N in soil leachates (n = 3) 5](#_Toc38441143)

# Methods

## Soil column setup


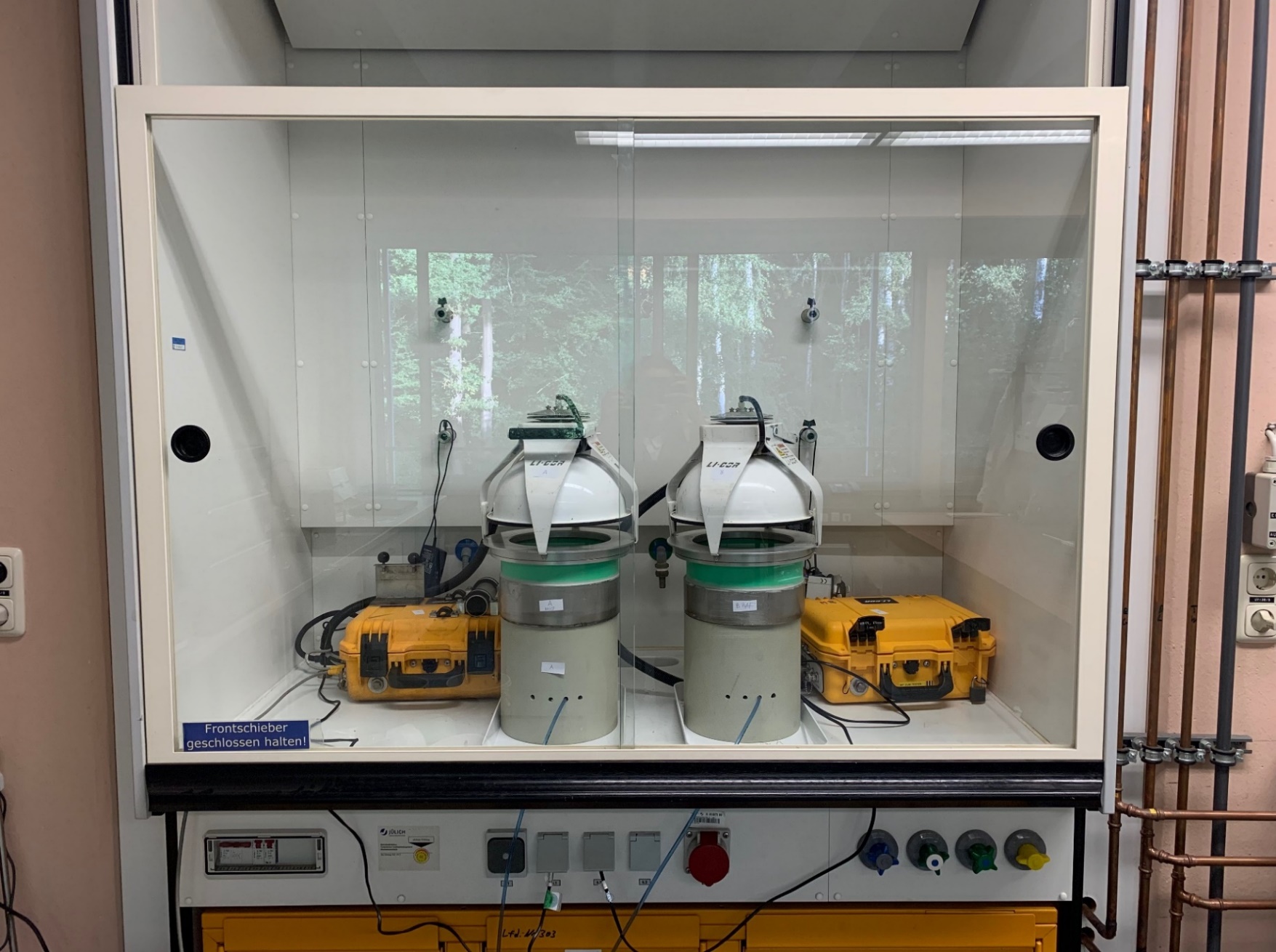


Chamber

CO_2_ analyser

Drainage

Column

Retainer


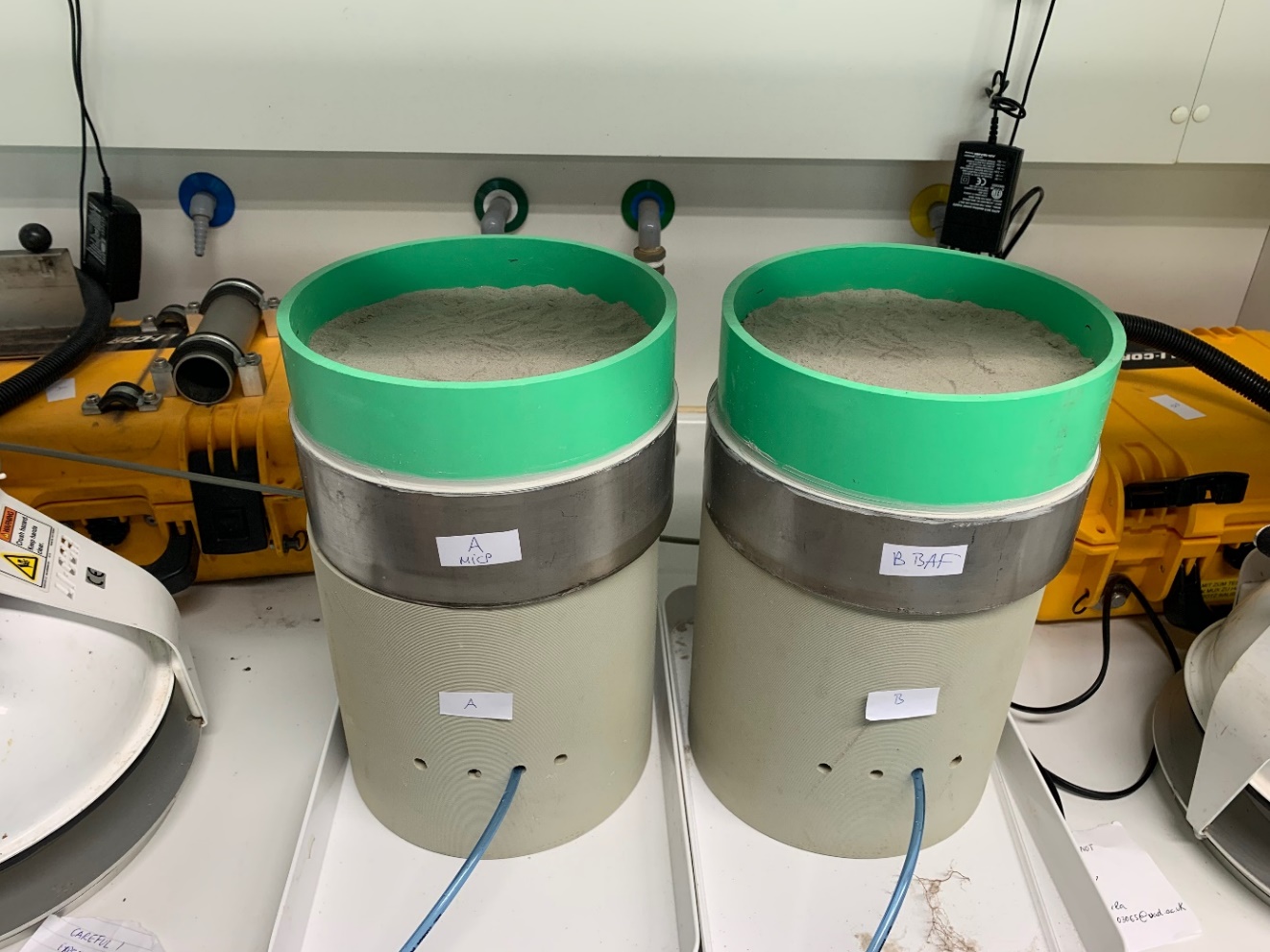


Figure S1 Soil column setup and measuring equipment

## Soil column experiment phases


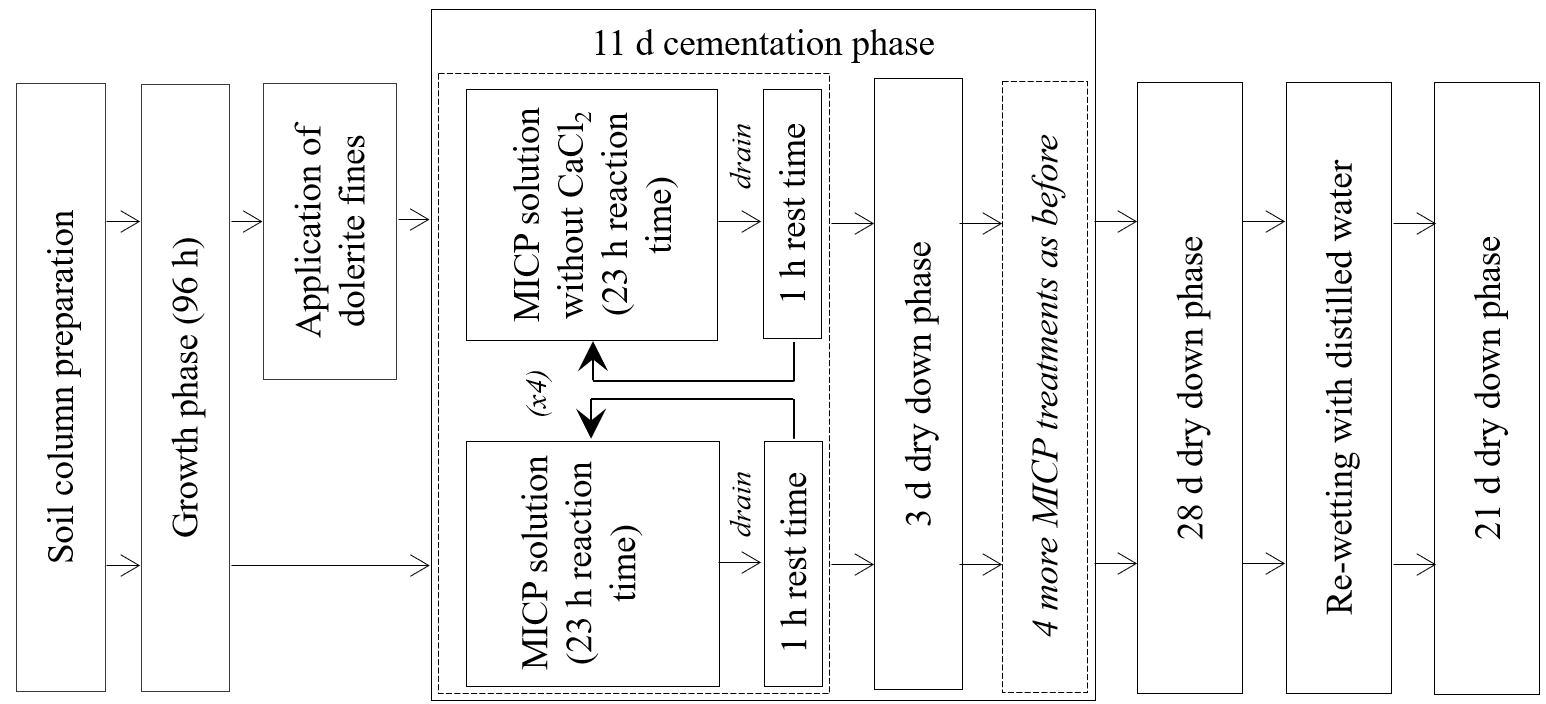


Figure S2 Scheme of the soil column experiment phases over the two months period.

## Soil sampling


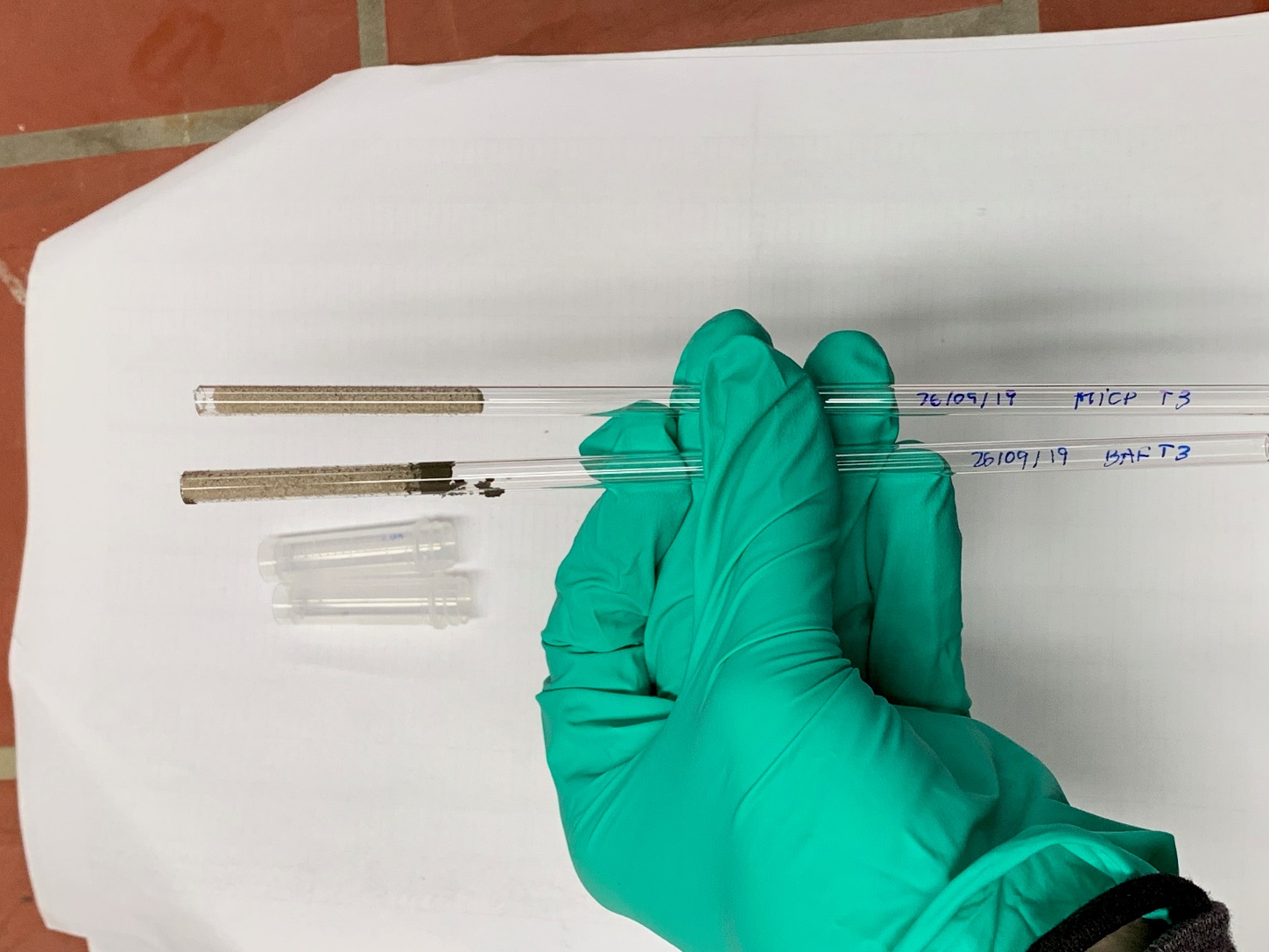


Soil

Dolerite from top layer

Figure S3 Soil sampling detail showing samples obtained for soil analysis and dolerite fines from top layer were sampled with soil

# Results

## ICP-OES analysis of soil leachates

Table S1 Elements ICP-OES (n =1). “t” stands for treatment. Analysis carried out by James Hutton Ltd., Aberdeen, UK. Units in mg/L.

| Treatment | Traditional MICP | | | Dolerite and MICP | | |
| --- | --- | --- | --- | --- | --- | --- |
| Element/Sampled | t1 | t8 | rewetting | t1 | t8 | rewetting |
| Al | 1.08 | 0.73 | 2 | 1.7 | 0.89 | 145.1 |
| B | <0.02 | <0.02 | 0.5 | 0.04 | <0.02 | 0.5 |
| Ba | 0.0532 | 0.0109 | 0.015 | 0.1321 | 0.0283 | 0.427 |
| Ca | 6.768 | 2.834 | 19.01 | 15.55 | 4.819 | 10.18 |
| Cd | 0.004 | 0.004 | <0.02 | 0.006 | 0.004 | <0.02 |
| Co | 0.014 | <0.002 | <0.01 | 0.011 | <0.002 | 0.03 |
| Cr | 0.009 | <0.008 | 0.09 | 0.026 | 0.011 | 0.36 |
| Cu | 0.284 | 0.398 | 4.37 | 0.737 | 0.143 | 6.97 |
| Fe | 0.436 | 0.301 | 1.56 | 1.138 | 0.655 | 78.47 |
| K | 67.97 | 58.43 | 80.4 | 69.14 | 58.05 | 26.8 |
| Mg | 2.364 | 1.772 | 2.29 | 2.41 | 5.419 | 9.57 |
| Mn | 0.05 | <0.02 | <0.1 | 0.16 | 0.15 | 1.2 |
| Mo | <0.04 | <0.04 | <0.2 | 0.05 | <0.04 | <0.2 |
| Na | 2895 | 2648 | 4922 | 3082 | 2490 | 553.2 |
| Ni | 0.35 | 0.1 | 0.9 | 0.44 | 0.07 | 1.1 |
| P | 0.93 | <0.04 | <0.2 | 2.31 | 0.44 | 1.2 |
| S | 3.4 | 1.4 | 5.1 | 13.1 | 2.8 | 9.4 |
| Se | 0.1 | <0.1 | <0.5 | 0.2 | 0.1 | <0.5 |
| Si | 6.32 | 6.36 | 24.3 | 220 | 9.03 | 183.5 |
| Sr | 0.056 | 0.039 | 0.1 | 0.062 | 0.118 | 0.4 |
| Ti | 0.0393 | 0.0228 | 0.02 | 0.1217 | 0.0354 | 1.79 |
| V | 0.0095 | 0.009 | <0.006 | 0.0188 | 0.0299 | 0.226 |
| W | <0.006 | <0.006 | <0.03 | <0.006 | <0.006 | <0.03 |
| Zn | 0.379 | 0.189 | 0.85 | 1.055 | 0.197 | 1.51 |

## Elemental C and N analysis and N compounds in soil leachates

Table S2 TOC, TN, urea-N, ammonium-N, nitrite-N and nitrate-N in soil leachates (n = 3). Units in mg/L.

| Treatment | | Traditional MICP | | | Dolerite and MICP | | |
| --- | --- | --- | --- | --- | --- | --- | --- |
| t | d | 1 | 8 | 37 | 1 | 8 | 37 |
| TOC | mg/L | 6382 | 4442 | 10510 | 7988 | 3962 | 2516 |
| TN | mg/L | 5324 | 2524 | 239 | 7764 | 1973 | 278 |
| Urea-N | mg/L | 802 | 219 | 0 | 1516 | 67 | 2100 |
| NH4-N | mg/L | 4180 | 2411 | 122 | 5168 | 1996 | 153 |
| NO3-N | mg/L | 14.4 | 32.7 | 14.8 | 14.4 | 11.8 | 42.2 |
| NO2-N | mg/L | 0.411 | 0.573 | 0.632 | 0.528 | 0.457 | 0.604 |
